# Sitting foot: posture dependent changes of volume, edema and perfusion of the foot. A prospective interventional study with 27 volunteers

**DOI:** 10.1186/s12891-024-07971-4

**Published:** 2024-10-29

**Authors:** Wolfgang Freund, Peter Wikstroem, Arthur P. Wunderlich, Uwe Schuetz, Meinrad Beer

**Affiliations:** 1 Diagnostic and Interventional Radiology, University Hospitals Ulm, Albert-Einstein-Allee 23, 89081 Ulm, Germany; 2 Urology, Hegau-Bodensee-Klinikum, Singen, Germany

**Keywords:** Posture, Sedentary lifestyle, Edema, Perfusion, Osteochondritis

## Abstract

**Background:**

Sitting is known to be bad for your cardiovascular health. We furthermore hypothesized that sitting posture will reduce perfusion of the foot and increase edema, possibly predisposing to disease like osteochondritis.

**Methods:**

We included 27 healthy volunteers and performed MRI measurements including arterial spin labelling (ASL) and intravoxel incoherent motion (IVIM) perfusion as well as short tau inversion recovery (STIR) edema measurement and 3D volumetry. After randomization, the elevation of one foot during the day was used as an intervention.

**Results:**

Intra- and interrater variability was 1–6%. ASL perfusion measurement was hindered by artifacts. IVIM perfusion showed no significant changes during supine measurements. Volumetry could demonstrate a highly significant (*p* = 0.00005) volume increase, while the intervention led to a significant (*p* = 0.0076) volume decrease during the day. However, the water content in STIR remained unchanged and the normalized (quotient bone/muscle) edema was reduced on the control side (*p* = 0.006) and increased on the intervention side (*p* = 0.01).

**Conclusions:**

Sitting all day leads to swelling of the healthy foot. Compensation in healthy subjects seems to prevent lasting perfusion changes or edema evolution in the bone despite an increase of muscle signal and volume increase. Thus, the etiology of osteochondritis needs further studies.

## Background

From the standpoint of evolution, the human foot is unique and has evolved to allow bipedal locomotion [1] and especially long distance running [2–5]. Survival of the fittest or fastest, however does no longer apply to our everyday sitting routine. Sitting too much has been shown to be deleterious to our health in many ways including arterial function [6] metabolic syndrome [7] and fracture risk [8].

Osteochondritis dissecans (OD) typically consists of a bony necrosis in a load bearing part of a joint with possible development of a free fragment damaging the joint. OD of the talus is a frequent affliction of unknown causes [9–12], often hitting adolescents [13] with an incidence of 4.7/100,000 in children [14] and 27/100,000 in army recruits [15]. OD develops mostly in adolescents in rounded joint surfaces with punctual load bearing and occurs even in animals [16, 17]. Known risk factors include obesity, smoking and diabetes [18], and even genetic factors are involved [19]. One theory holds that repeated microtraumata lead to impaired bone perfusion [12] which leads to necrosis and ultimately to the dissecating fragment. Thus, OD could be seen as a model of diseases caused by factors of vascular, metabolic and bone health. Due to our mostly sedentary occupation, physicians, and especially radiologists should worry about the possible consequences of sitting all day.

Own experience observing ultramarathon runners without development of OD excluded simple loading as a cause [3, 20]. Many of the patients with OD tali have no known risk factors and earlier reports hint at reduced microcirculation depending on the posture [21], possibly due to vasoconstrictor response [22]. Thus, we hypothesized that sedentary posture could lead to venous stasis, edema and decreased perfusion of the foot. Our study set out to non-invasively analyze the circadian and posture dependent changes of edema and perfusion of the foot and possible reversion of these effects by an intervention. To this end we performed MRI measurements before and after a sedentary work day. The intervention consisted of elevating one foot to prevent venous stasis.

## Methods

The STROBE checklist was used [23]. The subjects were recruited from healthy (no prior foot injury or disease of the feet stated on a survey) students and employees of the radiological department of our University hospital. Participation was on a day reserved for sitting work. To enhance participation, we asked permission of the department chair to investigate during routine work. Furthermore, we decided not to use radiation or contrast medium, so that the promising MRI techniques described below were utilized. After positive statement from our institutional ethics committee (Ethics Committee, Approval 229/13) following the stipulations on voluntarity and in accordance with the Declaration of Helsinki, healthy volunteers were recruited from the radiology department of our hospital. They gave written informed consent on the forms approved by the Ethics Committee. The 27 subjects were randomized regarding the side of intervention and then received the instruction to spend as much time as possible sitting. The intervention side foot was to be elevated on desk level with the aid of a special cushion, the foot in the normal position was named “control”. Movement and posture were documented every quarter of an hour, thereby obtaining precise data on posture without too much interference in work routine. The MRI image acquisition took place in the morning and after at least six hours of intervention. Representing the predominantly female workforce of our department, our study sample consisted of 20 female and 7 male subjects, their age ranging from 23 to 60 years with a mean of 38 years. 14 had the intervention on the right foot, 13 on the left.

The mean duration of the experiment was 6 h and 45 min (standard deviation SD 35.5 min). On average, subjects remained sitting in the experimental position (elevating the intervention side foot) for 4 h and 33 min (SD 69.3 min). They were sitting with both feet on the ground for 74.5 min (SD 63.2) and walked for 43.2 min (SD 29.7) or stood for 9.6 min (SD 14.6) with 3.9 min of other postures like lying down or cycling.

MRI scanning employed a 3T Magnetom Skyra (Siemens Healthineers) with a dedicated foot/ankle 16 channel coil. We took great care to use a standardized fitting approach where the foot was placed always with the medial side and hallux to the side of the coil and the foot was placed firmly against the sole of the coil. We performed arterial spin labeling (ASL) perfusion [24, 25], intravoxel incoherent motion (IVIM) perfusion [26] in the talus as well as measurement of volume changes of the foot with automated volumetry using volume rendering in an anatomical T1 weighted sequence and measurement of edema with short tau inversion recovery (STIR). The scanning protocol is listed in Table 1.

**Table 1:** Scanning parameters of the MR-sequences used

Scanning parameters	FLASH 3D	STIR	Diffusion	ASL 3D
B-values	n.a.	n.a.	0; 10; 20; 30; 40; 50; 80; 100; 200; 400; 800	n.a.
TR	1040 ms	4800 ms	2000	3000
TE	2.97 ms	39 ms	59	36.22
TI	n.a.	202 ms	n.a.	n.a.
FA	9°	150°	90°	180°
ETL	1	13	51	21
Resolution	1 × 1 mm	0.6 × 0.6 mm	3 × 3 mm	3 × 3 mm
Slice thickness	1 mm	3 mm	2.5 mm	3.27 mm

Abbreviations: FLASH fast low angle shot, 3D three dimensional, STIR short tau inversion recovery, ASL arterial spin labelling, TR time of repetition, TE time of echo, TI time of inversion, ETL echo train length, FA flip angle, n.a. not applicable

Measurements: Two raters (WF, PW) independently and blinded to the intervention side performed the measurements.

MRI data were transferred to a workstation using the program IntelliSpace version 8 (Philips).

**Fig. 1:**
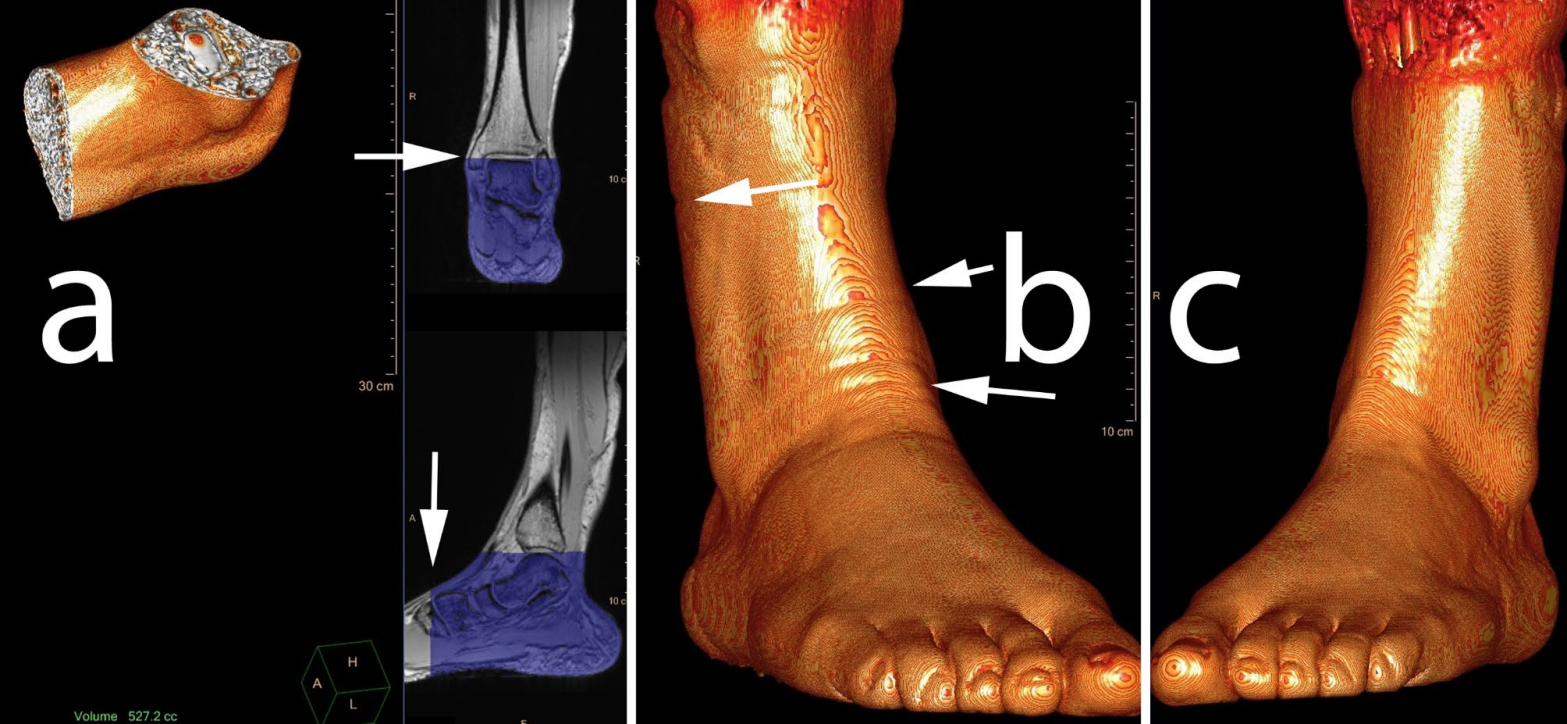
Volumetry using manually defined boundaries (**a**) and automatic volumetry. In the evening, the control side (**b**) shows swelling with visible indentions from the sock (arrows), while the intervention side (**c**) does not

The foot volume was measured semiautomatically with IntelliSpace performing volume rendering with the T1 3D data. After exclusion of all signal outside the volume defined proximally by the upper limit of the talus and anteriorly by the most distal limit of the os cuneiforme mediale, the volume of the foot was calculated by the program IntelliSpace (Fig. 1a). After the procedure, all calculation steps were erased and the procedure and measurement were repeated.

**Fig. 2:**
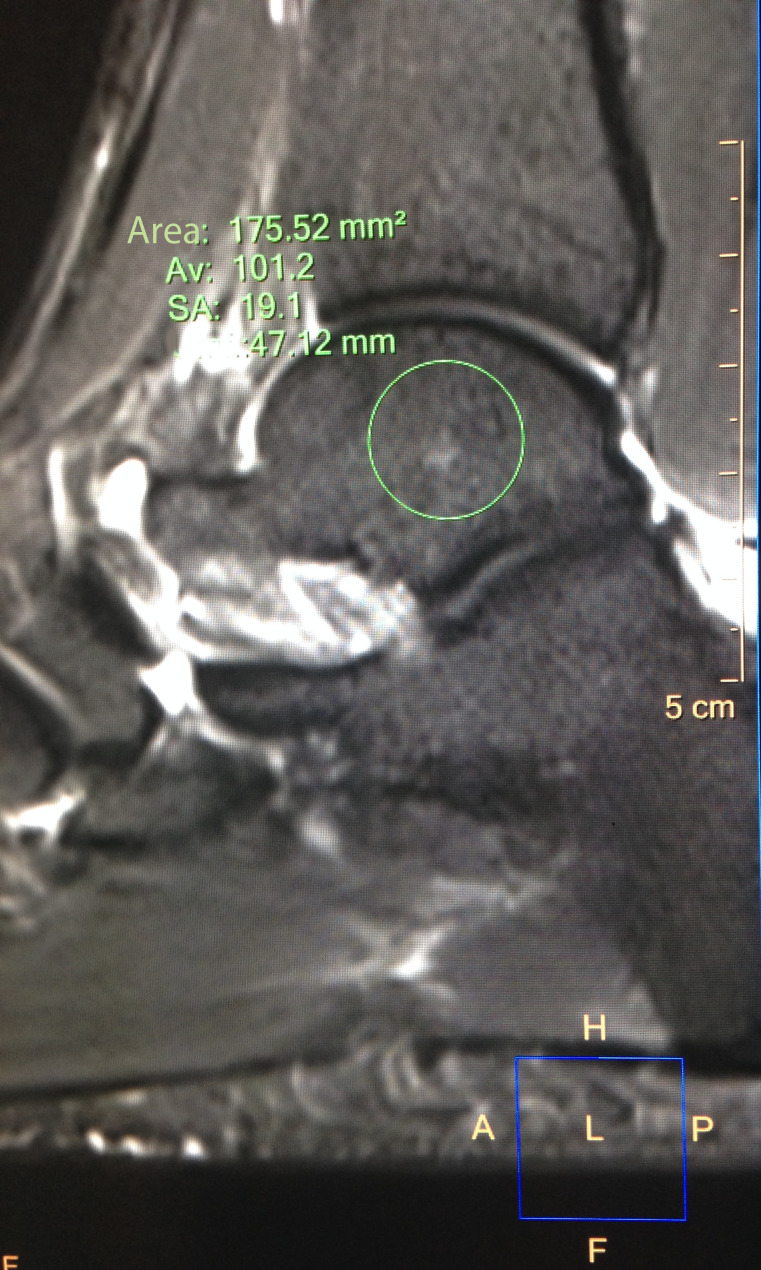
ROI placement for STIR SI measurements. The ROI was drawn centrally in the talus with a diameter of 15 mm (+/- 0.5 mm), avoiding visible vessels or artifacts, if possible. The resulting area is 165–189 mm^2^

STIR signal was obtained in two regions of interest (ROIs) (see Fig. 2) of 15 mm (+/- 0.5 mm) diameter each with an area of 165-189mm2, placed centrally in the talus (avoiding visible vessels or artifacts [27]). Two reference ROIs were placed in the flexor hallucis longus muscle (being the nearest large muscle). The reference ROIs in the muscle were done only by one reader (PW). To control for changes in general signal due to technical variations (e.g. coil and gradient temperature) the talus SI was referenced to muscle SI by calculation of the quotient SI talus/ SI muscle.

IVIM signal was obtained in parameter maps provided by the work in progress Program MR Body Diffusion Toolbox (Siemens Healthineers). The values for fractional perfusion were calculated for a ROI placed centrally in the talus (one rater PW).

ASL signal was measured twice in a region of interest (ROI) of 15 mm (+/- 0.5 mm) diameter each with an area of 165-189mm^2^ located centrally in the talus.

Statistical analysis: Data were fed into an Excel (Microsoft) spreadsheet and descriptive statistics and t-test and intraclass correlation ICC (using the real statistics resource pack 4.5 [28]) was performed. *P* < 0.05 was taken as a measure of significance. Interrater- as well as intrarater-differences were calculated.

## Results

**Fig. 3:**
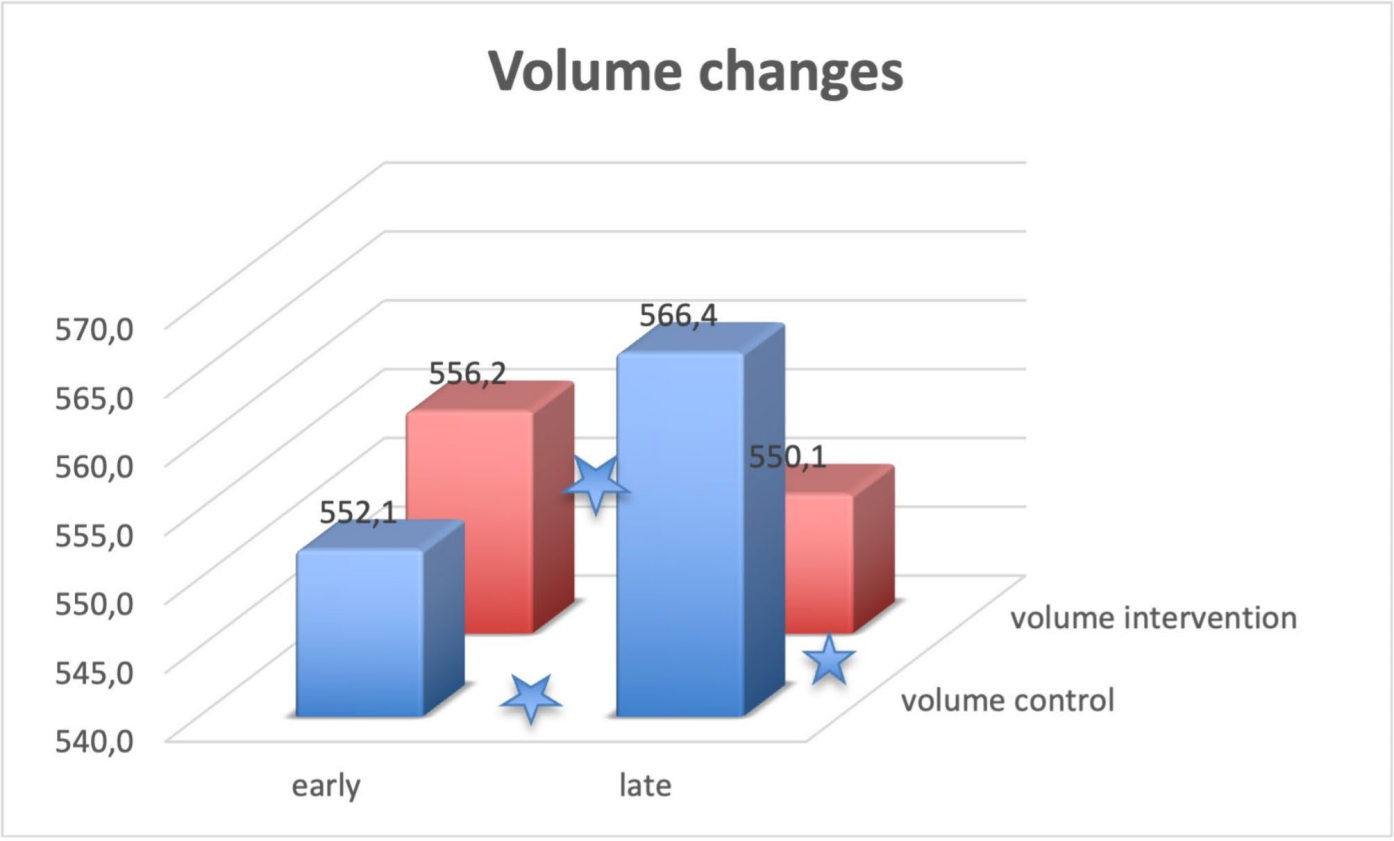
Volumetry measurements of the foot Volumes of the intervention side and control side are shown before and after the intervention. Asteriscs denote significant differences, see also Table 2

**Fig. 4:**
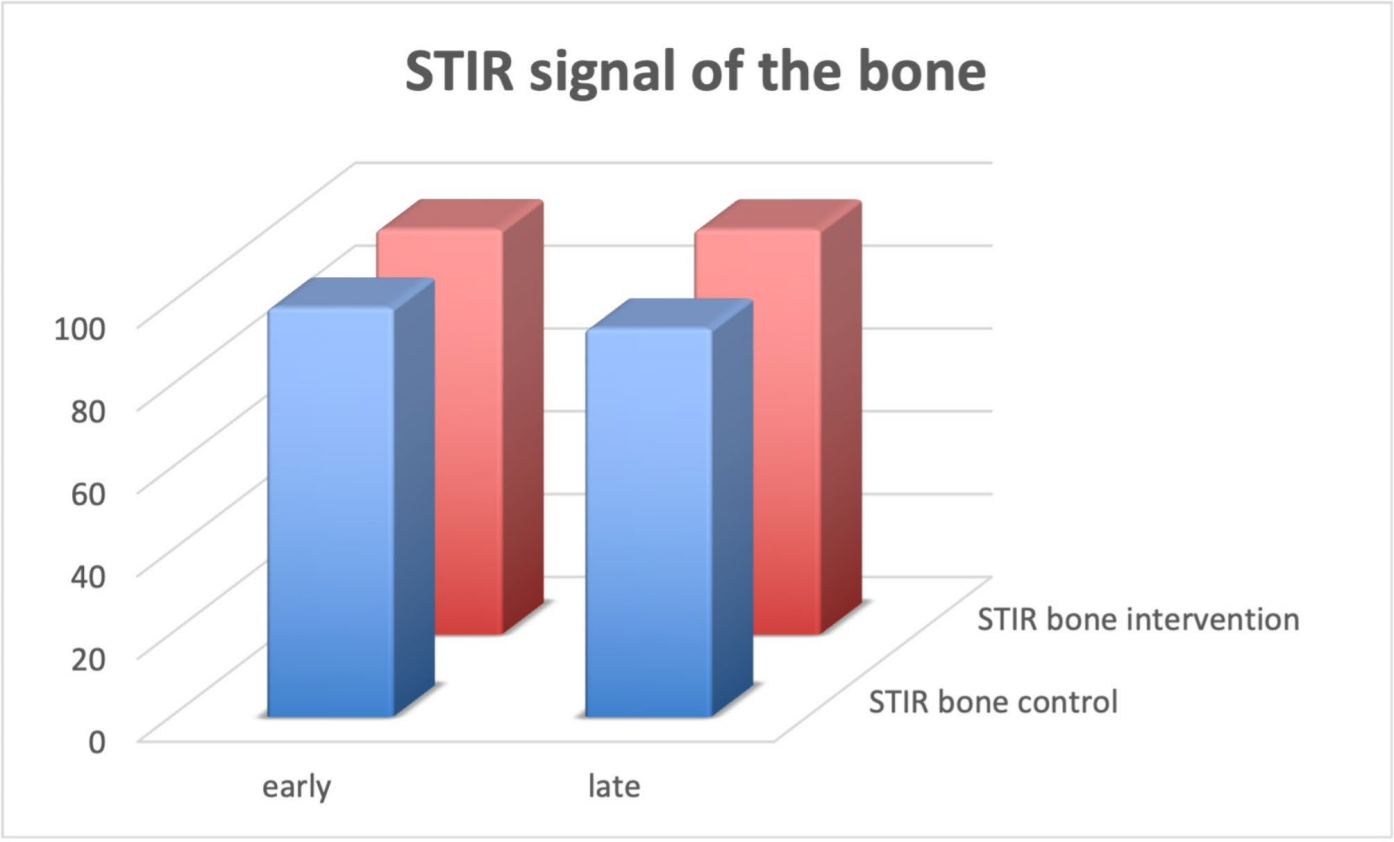
STIR signal measurements of the talus Signal intensity of the bone of the intervention side and control side are shown before and after the intervention. No significant differences, see also Table 2

**Fig. 5:**
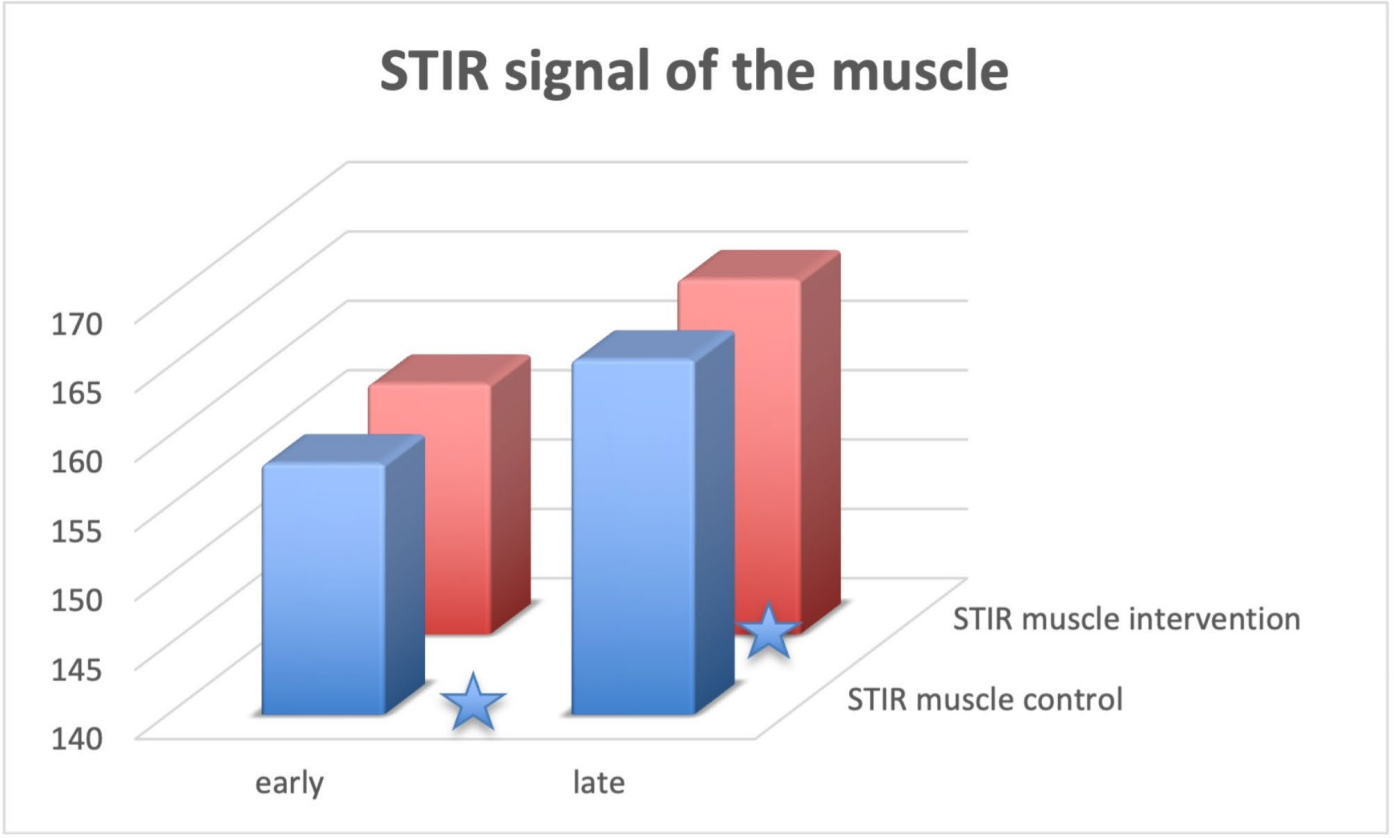
STIR signal measurements of a control area in the muscle Signal intensity of the intervention side and control side are given before and after the intervention. Asteriscs denote significant differences, see also Table 2

The resulting data are shown in Table 2, with Fig. 3 demonstrating volume changes, Fig. 4 STIR signal measurements of the bone (talus) and Fig. 5 STIR signal measurements of the control area in the muscle.

**Table 2:** Mean measurement results. The part of the table referring to the measurements in the morning (early) is bright, the evening measurements are in the shaded part of the table, significant results are shown in bold letters

Measurements Mean (SD)	Interv. side	control side	*p* side difference	Interv. side	control side	*p* side difference	*p* difference late-early
Time point	early			late			intervention
IVIM_FP (%)	22.9 (5.6)	22.0 (4.6)	0.53	21.6 (6.9)	21.5 (3.0)	0.94	0.22
Volume in ml	556.2 (107.2)	552.1 (107.2)	0.2	550.1 (109.3)	566.4 (108.5)	**0.00005**	**0.0076**
STIR SI talus	97.9 (16.8)	98.8 (17.1)	0.63	97.8 (22.2)	93.8 (23.6)	0.12	0.12
STIR SI muscle	158.1 (19.5)	158.1 (24.3)	0.99	141.7 (17.8)	165.6 (32.5)	**0.001**	**0.0003**
Rel SI (talus/muscle)	0.63	0.64	0.8	0.69	0.58	**0.0003**	**0.006**

Abbreviations: Interv. Intervention (elevated foot), IVIM_FP intravoxel incoherent motion measurement fractional perfusion signal, STIR short tau inversion recovery, SI signal intensity, Rel SI relative signal intensity computed by division of SI talus/SI muscle

**Fig. 6:**
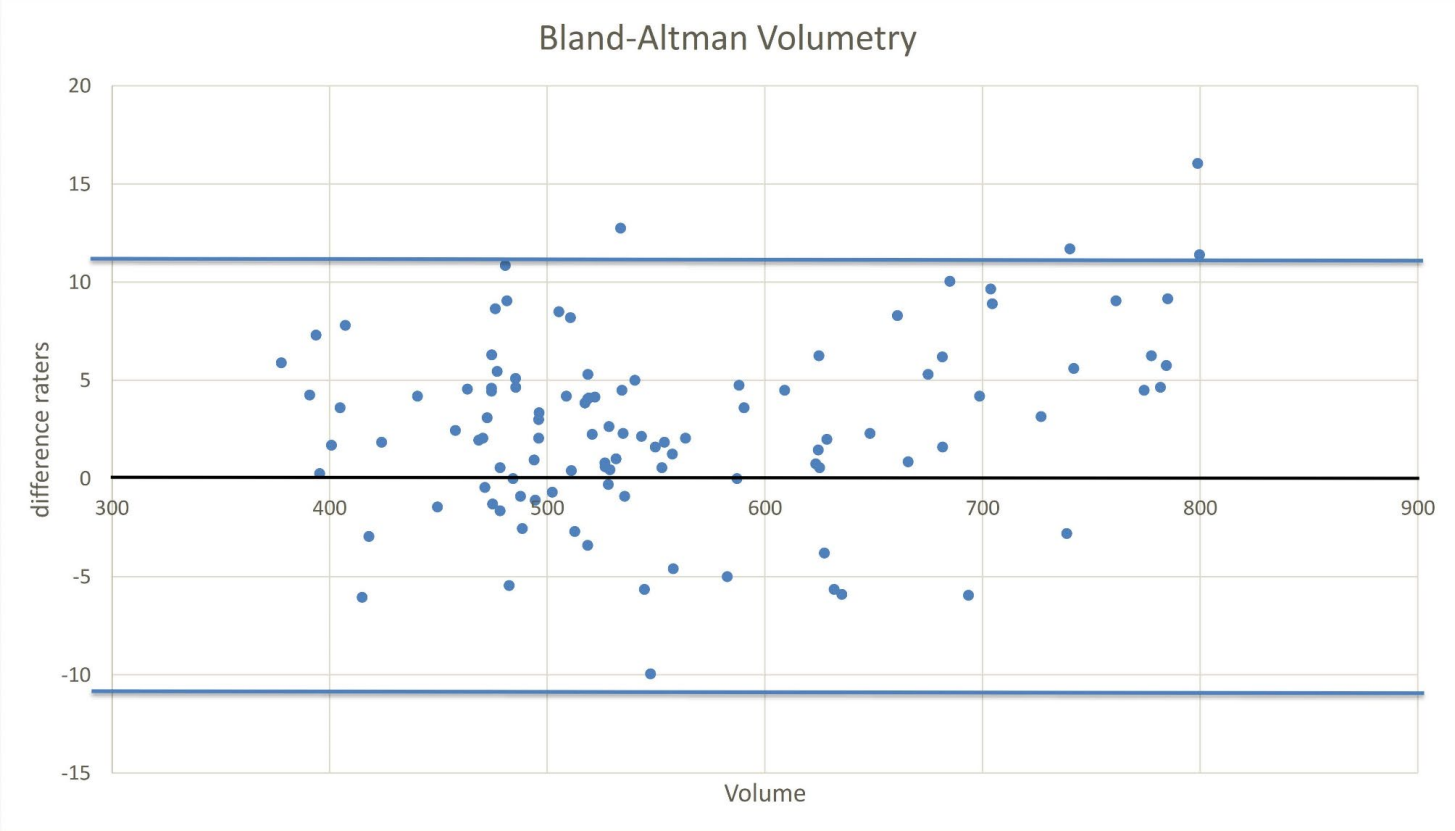
Bland Altman plot of volumetry measurements On the x-axis the volume measurements are listed, on the y-axis the difference between the two readers is shown

**Fig. 7:**
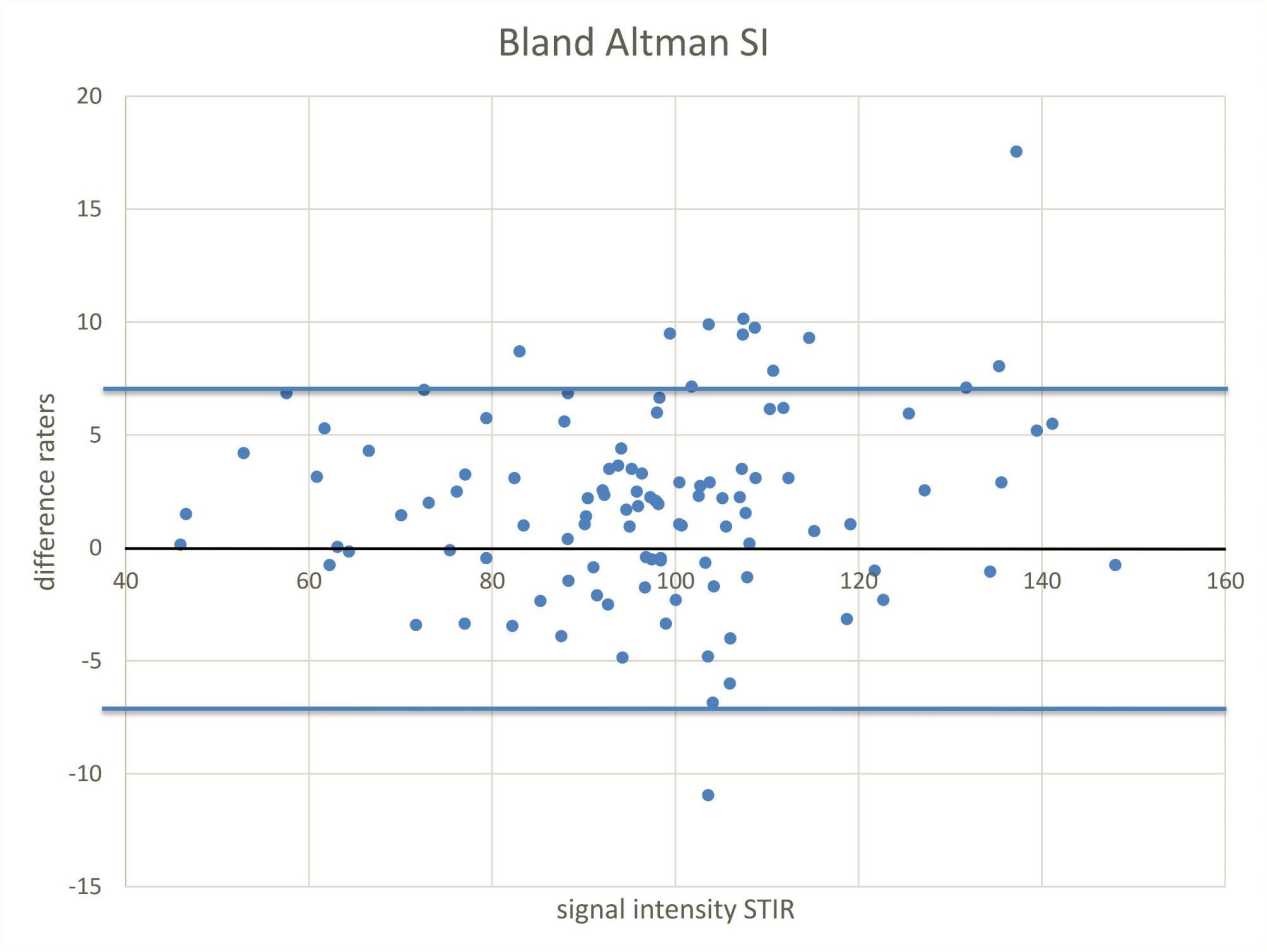
Bland Altman plot of SI measurements in the talus On the x-axis the SI measurements are listed, on the y-axis the difference between the two readers is shown

Regarding the reliability of the edema and volume measurements, intra- and interrater differences were calculated. For measurements of STIR signal intensity (SI) of the talus, intrarater ICC was 0.968 and interrater ICC was 0.956. In the semiautomatic volumetry, intrarater as well as interrater differed by less than 1%, resulting in intrarater ICC of 0.997 and interrater ICC 0.998 (Figs. 6 and 7 show the Bland Altman plots of the measurements).

Regarding the changes of STIR measurements during the day (see Fig. 4 + 5), SI in the reference muscle increased significantly on the control side (*p* = 0.036) and decreased on the intervention side (*p* = 0.0001). At the same time, talus STIR SI decreased 5% on the control side (n.s.), but remained stable on the intervention side (significant difference between sides *p* = 0.02). Similarly, the quotient SI talus/SI muscle was 19% higher on the intervention side in the evening (*p* = 0.01), while in the morning, there was no significant side difference (*p* = 0.8).

Regarding the volumetry, the control side (normal sitting position) showed a swelling of 14 ml during the day (corresponding to 3%, *p* = 0.008) while the intervention side showed a volume reduction of 6ml (*p* = 0.00005), see Figs. 1 and 3; Table 2.

**Fig. 8:**
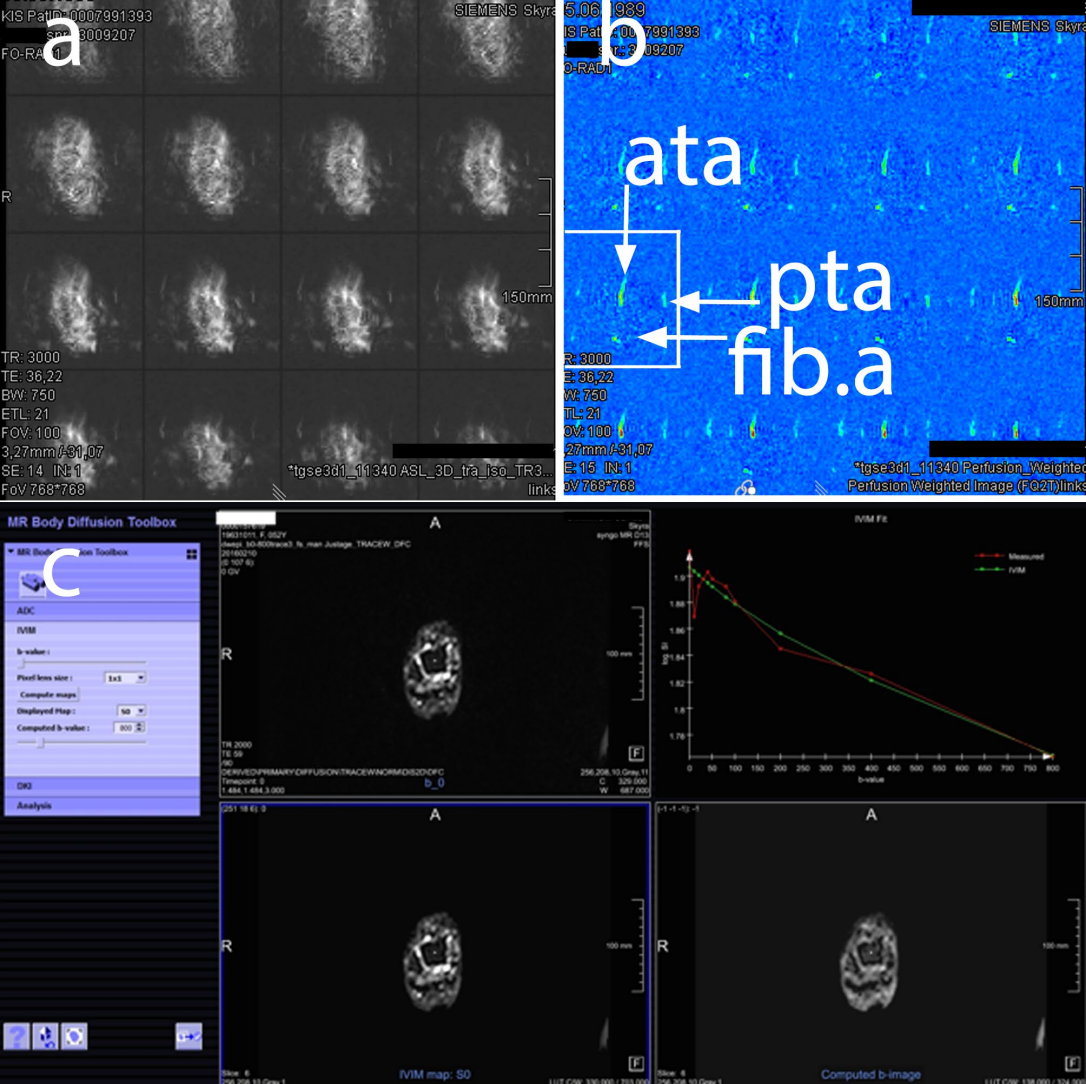
Results from ASL (**a**, **b**) and IVIM (**c**) measurements ASL scan (**a**) and ASL perfusion map (**b**) showing high arterial signal in the anterior tibial artery ata, posterior tibial artery pta and fibular artery fib.a. IVIM (**c**) is computed by analyzing the different b-value maps, resulting in an estimate of the perfusion

Regarding the perfusion measurements, ASL measurements had shown the feasibility of the method in preliminary tests. In the main study, however, there were too many artifacts to measure the perfusion. An exemplary ASL examination is shown (Fig. 8a + b), however due to massive artifacts, no results are presented.

In the IVIM measurements, fractional perfusion could be calculated. However, there was no statistically significant change in perfusion between the early and late measurements and between the sides (intervention = elevated or control = normal posture). The standard deviation of the measurements was quite high. See Table 2; Fig. 8c.

## Discussion

Our volumetric and STIR measurements of the talus showed highly reproducible results indicating significant posture dependent volume and SI changes of the foot during the day that will be discussed subsequently. IVIM measurements showed no significant effect on bone perfusion. Despite hopeful results in preliminary experiments, ASL could not be used to reliably measure perfusion of the talus.

Our volume measurements (see Figs. 1 and 3) were performed with intra- and interrater differences of less than 1%. The ICC values show excellent reliability [29].On the control side (normal sitting position), we were able to measure a relevant volume increase of the foot. Clinically, this corresponds to the swelling of the foot, and is due to an increase in volume as a result of an increased venous stagnation on the control side. Increased elevation of the foot throughout the day prevents or even reverses the swelling. This result confirms investigations on occupational safety and work ergonomics in the relevant occupational medical literature [30].

Corresponding beneficial effects had been shown for intermittent negative pressure that seemingly enhances macro- and microcirculation [31].

Our STIR SI measurements of the talus showed excellent reliability with low inter- and intrarater differences of 2–4% and matching high ICC values. In line with the volumetric results, the intramuscular STIR signal also shows a significant increase on the control side and decreased on the intervention side. These results are in line with older reports on elevation of the limb to decrease intramuscular pressure [32].

Contrarily, and surprisingly, the intraosseous STIR SI showed no significant change on both sides, and the SI ratio of talus / muscle increased on the intervention side and decreased on the control side. The elevation of the foot does not seem to affect the intraosseous SI in the STIR sequence. Compared to the morning exam, intraosseous SI did not increase on the control side, but decreased somewhat, but not significantly: how do we explain this?

As STIR signal is mostly dependent on water content of the tissues [33] and is very sensitive to detect bone edema [34], our results show that venous stasis did not lead to an increase of intraosseous free water content like in soft- or muscle tissues. Further verification by using T1-weighted contrast-enhanced fat-suppressed spin-echo MR imaging are not necessary in this regard, because both sequences demonstrate almost identical imaging patterns, and diagnoses determined with these findings show little difference [35].

As there are various causes of bone marrow edema in the ankle and hindfoot [36], simple sitting in a chair seems not to be one of them. Several edema preventing mechanisms are known [37]: Vascular mechanisms, modifying capillary pressure or interstitial fluid pressure. As the interstitial fluid pressure in bone marrow is higher and the extracellular matrix is denser than in muscle or soft tissues, the third mechanism may play a central role [38], but the complex interactions of mechanical, hormonal and metabolic influencing factors in osseous blood vessels and flow are only partially understood till now [39–42]. Furthermore, existing literature has shown, that elevation of the foot in the context of severely damaged circulation such as diabetic foot syndrome may be detrimental to wound healing [43].

IVIM measurements can be analyzed to provide an estimate of parenchyma perfusion [26, 44]. Lowering the dependent foot decreases blood flow by 79% [45] probably centrally as well as locally mediated. Our results do not show any change in perfusion in our healthy volunteers, so we must assume that our experimental setting of performing perfusion measurements in the supine position has been taken place too late, because readaptation and compensatory vasopressor effects may have already occurred.

## Strengths and limitations

Our study could use a modern multiparametric MRI setup to measure noninvasively the effects of prolonged sitting. The sample of subjects represents a typical workforce for a radiology department. The results are an important addition to the known facts of the changes occurring during sitting.

However, we could not measure perfusion using ASL. This might have been attributable to low perfusion of the talus compared to brain tissue, where ASL is routinely used [44]. We could not detect perfusion changes in the supine position, but have to suspect intermittent short-term changes that were reversed too fast to be detectable. MRI measurements while still sitting are not possible in clinical scanners with horizontal bore, open systems exist only with 1,5 T strength.

Since our perfusion results were obtained only with the method IVIM, we could not reproduce them with another method. Thus these data must be seen as exploratory and would benefit from independent reproduction.

Furthermore, our results must be seen as preliminary, due to the small sample size and our choice of healthy medical staff as subjects. Also the setting was optimized to gain maximal effects. We are aware that continuous elevation is not feasible, as well as motionless sitting is not desirable in the workspace.

## Conclusions

Returning to our research hypothesis that the sedentary lifestyle could be responsible for diminished perfusion of the talus and thus increase the risk for osteochondritis, we have to concede that we could reliably document the swelling of the dependent foot and could show that foot elevation is successful in preventing the swelling, but we could find no proof of persisting diminished talus perfusion or increased water content after a day´s worth of sitting. Thus, radiologists (as well as other sitting professions) should worry about too much sitting because of its effect on bone density [46] or the general deleterious effects on our cardiovascular health [47], but don´t have to worry about osteochondritis. On the other side, the swelling effect can reliably be prevented by foot elevation. Further research could show how much elevation time is needed to prevent the swelling caused by prolonged sitting.

However, further research in risk groups or with pre-existing conditions is warranted.

## Data Availability

Data generated in this study are available from the corresponding author upon request.

## Abbreviations

OD: osteochondritis dissecans; A disease affecting the bones of weight bearing joints; possibly caused by reduced perfusion
ASL: arterial spin labelling: A MRI method to non invasively measure perfusion.
ASL: arterial spin labelling: A MRI method to non invasively measure perfusion.
IVIM: intravoxel incoherent motion: Another MRI method to non invasively measure perfusion.
STIR: short tau inversion recovery: A MRI technique to assess fluid content (edema) of tissue.
ICC: intraclass correlation: A statistical method to assess the reliability of measurements.
SD: standard deviation: A statistical measure for the amount of variation of results.
SI: signal intensity: The readout of the MRI measurement.
